# The analysis of transcriptomic signature of TNBC—searching for the potential RNA-based predictive biomarkers to determine the chemotherapy sensitivity

**DOI:** 10.1007/s13353-024-00876-x

**Published:** 2024-05-09

**Authors:** Stanislaw Supplitt, Pawel Karpinski, Maria Sasiadek, Lukasz Laczmanski, Dorota Kujawa, Rafal Matkowski, Piotr Kasprzak, Mariola Abrahamowska, Adam Maciejczyk, Ewelina Iwaneczko, Izabela Laczmanska

**Affiliations:** 1 Lower Silesian Oncology, Pulmonology and Hematology Center, Hirszfelda Sq. 12, 53-413 Wroclaw, Poland; 2 Department of Genetics, Wroclaw Medical University, Marcinkowskiego 1, 50-368 Wroclaw, Poland; 3 Laboratory of Genomics and Bioinformatics, Ludwik Hirszfeld Institute of Immunology and Experimental Therapy, Polish Academy of Sciences, Wroclaw, Poland; 4 Department of Oncology, Wroclaw Medical University, Hirszfelda 12, 53-413 Wroclaw, Poland

**Keywords:** Transcriptome, RNA-seq, TNBC, Breast cancer, Doxorubicin, Paclitaxel

## Abstract

Neoadjuvant chemotherapy is the foundation treatment for triple-negative breast cancer (TNBC) and frequently results in pathological complete response (pCR). However, there are large differences in clinical response and survival after neoadjuvant chemotherapy of TNBC patients. The aim was to identify genes whose expression significantly associates with the efficacy of neoadjuvant chemotherapy in patients with TNBC. Transcriptomes of 46 formalin-fixed paraffin-embedded (FFPE) tumor samples from TNBC patients were analyzed by RNA-seq by comparing 26 TNBCs with pCR *versus* 20 TNBCs with pathological partial remission (pPR). Subsequently, we narrowed down the list of genes to those that strongly correlated with drug sensitivity of 63 breast cancer cell lines based on Dependency Map Consortium data re-analysis. Furthermore, the list of genes was limited to those presenting specific expression in breast tumor cells as revealed in three large published single-cell RNA-seq breast cancer datasets. Finally, we analyzed which of the selected genes were significantly associated with overall survival (OS) in TNBC TCGA dataset. A total of 105 genes were significantly differentially expressed in comparison between pPR versus pCR. As revealed by PLSR analysis in breast cancer cell lines, out of 105 deregulated genes, 42 were associated with sensitivity to docetaxel, doxorubicin, paclitaxel, and/or cyclophosphamide. We found that 24 out of 42 sensitivity-associated genes displayed intermediate or strong expression in breast malignant cells using single-cell RNAseq re-analysis. Finally, 10 out of 24 genes were significantly associated with overall survival in TNBC TCGA dataset. Our RNA-seq-based findings suggest that there might be transcriptomic signature consisted of 24 genes specifically expressed in tumor malignant cells for predicting neoadjuvant response in FFPE samples from TNBC patients prior to treatment initiation. Additionally, nine out of 24 genes were potential survival predictors in TNBC. This group of 24 genes should be further investigated for its potential to be translated into a predictive test(s).

**Supplementary Information:**

The online version contains supplementary material available at 10.1007/s13353-024-00876-x.

## Background

Breast cancer (BC) is known up to date as a highly heterogeneous disease presenting not only a wide spectrum of pathological features or clinical symptoms, but also a large variety of alterations on genetic, epigenetic, and transcriptomic levels (Lüönd et al. Jul. 2021). Traditional classification of breast cancer includes biological characteristics such as tumor size, lymph node involvement, histological grade, patient’s age, expression of estrogen receptors (ER), progesterone receptors (PR), and human epidermal growth factor receptor 2 (HER2) (Yersal and Barutca Aug. 2014). The BC’s subtype of particular note is triple-negative breast cancer (TNBC) which is characterized by the lack of expression of three main therapeutic targets (ER, PR, HER2). It accounts for 15–20% of all BC cases and is more aggressive, with faster growth rate, higher risk of metastasis, and recurrence risk and thus worse prognosis than BCs with positive hormone receptors status (Cserni, et al. 2021). Due to its special molecular phenotype (lost expression of receptors), TNBC is sensitive to neither endocrine therapy nor molecular targeted therapy. In such cases, chemotherapy is the main systemic treatment, but the efficacy of conventional postoperative adjuvant chemoradiotherapy for TNBC is poor (Yin et al. Jun. 2020; Zhang et al. Mar. 2022; Cortazar et al. Jul. 2014).

The studies of recent years have shown that the utility of neoadjuvant chemotherapy in the treatment of TNBC results in a significantly higher pathological complete response (pCR) than in hormone receptor-positive BC cases (Holanek, et al. 2021). Achieving pCR is a predictor of better long-term treatment outcomes (Minckwitz et al. Oct. 2013). The current guidelines recommend using combination regimens based on taxane, anthracycline, cyclophosphamide, cisplatin, and fluorouracil. Standard neoadjuvant strategy includes regimens with anthracycline (epirubicin/doxorubicin) + cyclofosfamide (AC) followed by cycles with taxanes (docetaxel/paclitaxel). This scheme presents significantly better pCR rates in patients with TNBC compared to non-TNBC (51.2% vs. 12%) (Lee 2023; Wang and ほか, 2009). Nevertheless, the controversy arises from the fact that TNBC is a highly heterogeneous disease, and different sensitivity to commonly used agents is observed (Bai et al. Jan. 2021).

According to current knowledge, every cancer diagnosis is unique. The traditional classification of BC, based on histological appearance of tumors, presents limitations in terms of personalized treatment strategies—do not refer to complex genetic alterations underlying biological events in cancer progression (Yersal and Barutca Aug. 2014). Tumors with similar pathological presentations may have different behaviors, e.g., proliferative potential. In research and current practice, various attempts are made to distinguish molecular subtypes of different cancer in order to find its clinical implications. In terms of TNBC and other breast cancer subtypes, Ki67 index is used as the proliferation biomarker of aggressive, metastatic disease with poor outcome (Arafah et al. 2021). However, Ki67 assessment in immunohistochemistry presents some limitations, including low intra- and inter-laboratory reproducibility, inconsistent selection of antibodies for testing, potential problems resulting from tumor heterogeneity, and variation in laboratory reports due to different methods of cell counting (Dowsett et al. Nov. 2011). One of the first insights into TNBCs molecular heterogeneity was the observation of six distinct TNBC molecular subtypes by Lehhman et al. who distinguished including two basal-like (BL1 and BL2), an immunomodulatory (IM), a mesenchymal (M), a mesenchymal stem–like (MSL), and a luminal androgen receptor (LAR) subtype (Lehmann et al. Jul. 2011). Subsequently, refined classifications provided confirmation for four to two stable TNBCs with the most prominent biological and clinical evidence for existence of LAR and non-LAR subtypes (Chen et al. 2012; Yu et al. 2022; Thompson et al. 2022a). Importantly, several studies clearly showed that LAR patients display a significantly poorer response rate to neoadjuvant chemotherapy (Thompson, et al. 2022a). Thus, the success of major diagnostic and treatment challenges is dependent on defining specific TNBC subtypes and broad repertoire of biomarkers affecting personalized approach in TNBC patients (Balkenhol et al. Jun. 2020).

The molecular characterization of cancer tissues has become one of the key steps not only in cancer diagnostics, prognosis, and tailored therapy but also in searching for new molecular biomarkers and pathways (Pennock et al. 2019). A methodology which arose rapidly as a game-changer in transcriptomic analysis and the discovery of new biomarkers is whole tumor transcriptome analysis (whole RNA sequencing, RNA-seq). RNA-seq in contrast to genome sequencing allows analyzing not only nucleic acid sequence but also RNA expression level, new RNA molecules’ sequences (splice variants, chimeric genes, fusions), and non-coding RNAs. This makes RNA-seq an excellent and powerful technique for molecular analysis of cancer cells (Barrón-Gallardo et al. 2022). Moreover, RNA-seq technology may be performed using formalin fixed paraffin embedded (FFPE)–derived RNA. It allows selecting and using FFPE cancer tissues previously stored in biorepositories from patients with known clinical history (Pennock et al. 2019).

The main goal of our study was to evaluate using RNA-seq the transcriptomic patterns in FFPE cancer tissues derived from two groups of TNBC patients who were resistant and sensitive to AC neoadjuvant chemotherapy and, consequently, to identify the differentially expressed genes for their potential use as patient-tailored biomarkers.

## Materials

### Patient recruitment and selection criteria

Adult female patients with a diagnosis of TNBC, accepted to receive systemic neoadjuvant chemotherapy, were qualified for this study. Clinical exclusion criteria comprise disease with distant metastases (M1 and higher), hereditary breast cancer (BRCA-related disease), and patients previously treated against another cancer. Moreover, cases of insufficient biopsy tissue for further pathological or RNA analysis were excluded.

### Ethic statement

All patients signed an informed consent form before the genetic test. Approval was granted by the Ethics Committee of Wroclaw Medical University (No. 611/2019). All patients were diagnosed and treated in Breast Unit, Lower Silesian Oncology, Pulmonology and Hematology Center in Wroclaw, Poland. All breast cancer samples used in the study were taken only as a part of the patients’ diagnostic and therapeutic schemes. All procedures performed in this study were in accordance with the principles for medical research of the 1964 Declaration of Helsinki and its later amendments or comparable ethical standards.

### Treatment plan and study design

**Scheme 1:**
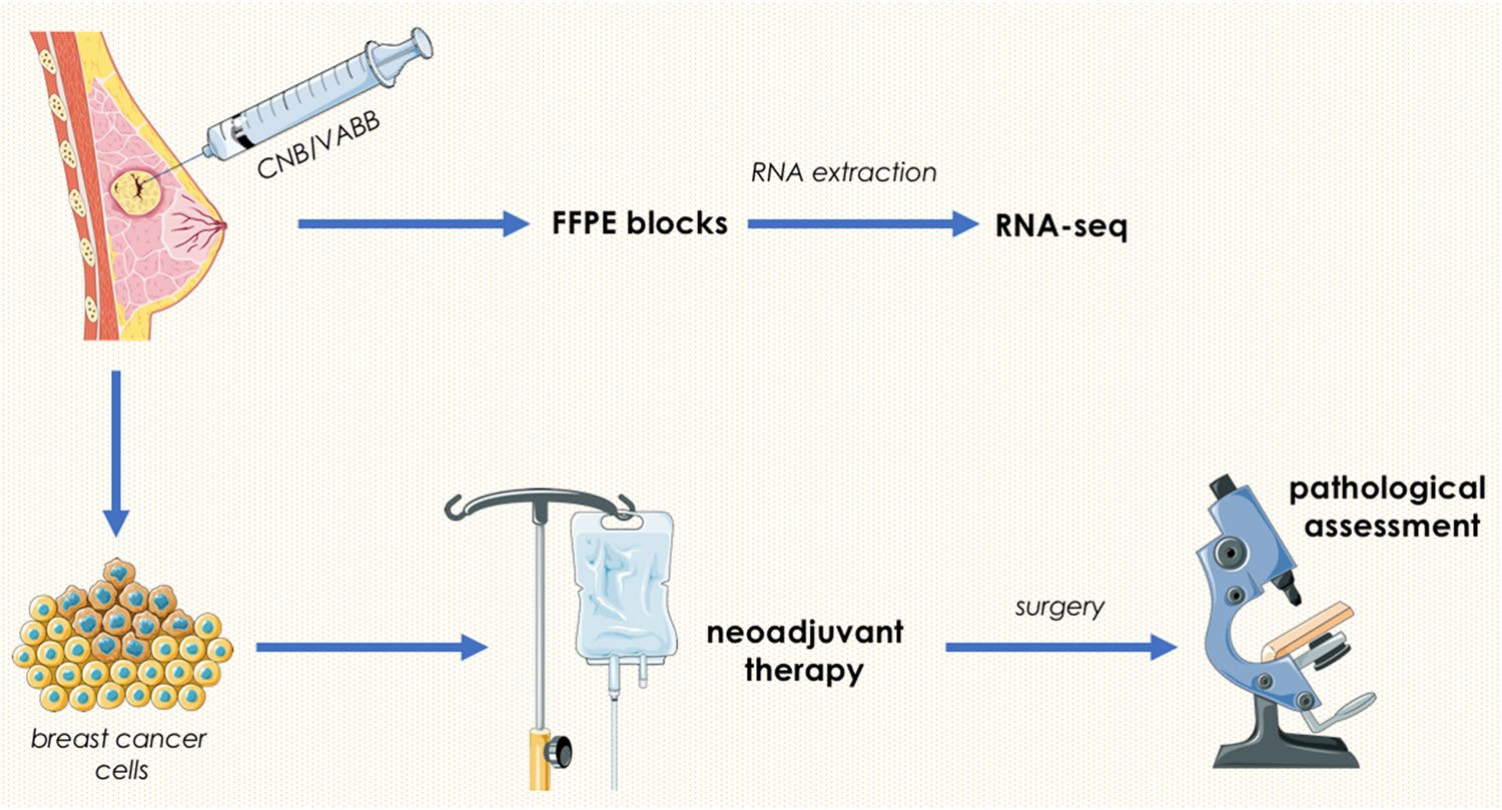
Treatment plan and related study design. Based on Smart Servier Medical Art (www.smart.servier.com)

Core needle biopsy and vacuum-assisted breast biopsy (CNB and VABB) breast samples, obtained from 46 TNBC-diagnosed patients (2018–2021) qualified to receive neoadjuvant therapy, were taken in FFPE blocks for RNA extraction and analysis. At first, the biopsies were taken before the AC chemotherapy onset. Neoadjuvant chemotherapy started with doxorubicin (at the dose of 60 mg/m^2^) with cyclophosphamide (600 mg/m^2^) cycled every 14–21 days for 4 cycles, followed every week by maximum 12 cycles of paclitaxel (80 mg/m^2^) or docetaxel (75 mg/m^2^). After chemotherapy, the breast samples were taken during surgery to assess the pathological response to treatment (Scheme 1). Patients with (pCR) were assigned to the sensitive group, while cases of pathologic partial response (pPR) and disease progression (PD) were set in the resistant group (reduced response group—RR). Pathological complete response (pCR) was defined as disappearance of all invasive cancer tissue in the resected breast specimen, as well as in all sampled regional lymph nodes after completion of neoadjuvant chemotherapy.

### External data acquisition

We obtained Rsubread preprocessed breast cancer TCGA data from Rahman et al. that included 1112 primary breast tumor samples (Rahman et al. May 2015). Survival data for TCGA breast cancer cases was obtained from Liu et al. (Liu et al. Apr. 2018). One hundred twenty-three triple-negative breast cancer (TNBC) cases from TCGA were selected based on data published by Thompson et al. (Thompson, et al. 2022b). Gene expression data and drug sensitivity for 63 breast cancer cell lines were obtained from Dependency Map (DepMap) Consortium (Ghandi et al. May 2019). We used TISH2 server to inspect selected gene expression compartment and gene expression values in breast cancer single-cell RNA-seq (sc-RNA-seq) experiments (Sun et al. Jan. 2021). We considered three datasets, and each included data for primary breast tumors in more than 10 patients (GSE176078, EMTAB8107, and GSE161529) (Wu et al. 2021; Qian et al. 2020; Pal et al. 2021).

## Methods

The pathologist’s role was the selection of the most representative breast cancer tissue sections containing at least 30% of cancer cells.

Total RNA was isolated from formalin-fixed, paraffin-embedded (FFPE) breast cancer tissue sections using the RNeasy® FFPE Kit (QIAGEN) according to the manufacturer’s protocol.

After extraction, the purity of RNA was determined on the NanoPhotometer N60 (Implen).

### RNA-seq

The quality check of the RNA has been performed with the use of High Sensitivity RNA ScreenTape on TapeStation (Perlan). Library construction was performed using KAPA HyperPrep Kit with RiboErase (HMR) according to manufacturer protocol with technical note for degraded inputs and 25 ng^–1^ µg of purified total RNA.

In brief, preparation of libraries consisted of depletion of a human rRNA, fragmentation using heat and magnesium, first-strand cDNA synthesis using random priming, combined second-strand synthesis, and A-tailing, adapter ligation, library amplification.

The library’s concentration was measured with the fluorometric method (QuantiFluor dsDNA System, Promega), and the quality check of the libraries was performed using capillary electrophoresis (High Sensitivity D1000 ScreenTape System on Tape Station (Perlan).

Libraries were diluted to 4 nM in accordance with NextSeq System Denature and Dilute Libraries Guide (Illumina) and pooled. Paired-end sequencing was carried out using the NextSeq HighOutput Reagents (Illumina).

### RNA-seq data pre-processing

Reverse and forward reads were merged, and subsequently, quality filtering and trimming was performed in Rfastp Bioconductor package. Next, filtered FASTQ files were aligned by Rsubread version 2.8.1 R package to GENECODE Release 33 (GRCh38.p13) reference genome (Frankish et al. Jan. 2021). Next, featureCounts() of Rsubread function was used to summarize the gene level expression values as integer number (raw counts). Depending on use, RNA-seq gene-level data were transformed in various ways. For differential expression calculations, we used raw counts. For integration with TCGA breast cancer dataset, we used ComBat-seq batch correction followed by log2-counts per million (log-CPM) transformation and quantile normalization (Zhang et al. 2020). For all other analyses, raw counts were transformed in log2-transcripts per million (log-TPM).

#### Statistical methods

All analyses were performed in R/Bioconductor environment. After the gene expression levels were derived, unexpressed or lowly expressed genes were removed using *filterByExpr* function in edgeR package (Chen et al. 2016). The number of genes retained for testing was 24,237. We used quasi-likelihood negative binomial generalized log-linear model implemented in edgeR package to calculate differentially expressed (DE) genes between study subgroups. DE analysis was adjusted for batch variable. DE genes were defined as those genes with a FDR corrected *p* value less than 0.05 and logFC > 1.2.

To assess which DE genes derived above may be associated with selected drug resistance (docetaxel, doxorubicin, paclitaxel, and cyclophosphamide), we used partial least squares regression (PLSR) implemented in mixOmics package (Rohart et al. 2017). In brief, we collected data on gene expression and drug sensitivity (the area under the fitted dose response curve—AUC) for 63 breast cancer cell lines. After limiting gene expression data to DE genes revealed above, we defined gene expression matrix as predictor and AUC matrix as response. Results of PLSR were visualized by relevance associations network in mixOmics package (Rohart et al. 2017). We only considered absolute correlation values ≥ 0.4 obtained by network function in mixOmics.

Expression profiles of our samples were assigned to two TNBC subtypes recently proposed by Thompson et al. (Thompson et al. 2022b): luminal androgen receptor (LAR) and non-luminal androgen receptor (Non-LAR). In brief, we used TCGA data consisted of 123 TNBC cases with TNBC subtype assignment provided by Thompson et al. (Thompson, et al. 2022b) to select set of discriminating genes between LAR and non-LAR subtypes. We used nearest template prediction (NTP) classifier as feature selection, classification, and prediction tool. TCGA dataset was used as train NTP (Eide et al. 2017; Hoshida 2010). Prediction confidence was assessed based on the distance of the null-distribution, estimated from 1000 permutation tests. FDR < 0.05 was used to correct the set of prediction confidence *p* values for multiple hypothesis testing. Survminer package was used to calculate univariate Cox proportional hazards model for analysis of association of expression of selected genes with overall survival in TCGA breast cancer samples limited to triple-negative cases. Cut points for gene expression values to define high/low expression levels were estimated by use of maximally selected rank statistics. Survival curves were drawn in Survminer package.

## Results

Basic patient characteristics are provided in Table 1. Using NTP classifier trained on TNBC TCGA data using signature composed of 390 genes, we identified 13 LAR and 31 non-LAR tumors in our dataset. Two out of 46 samples showed low prediction confidence (FDR ≥ 0.05) and were set to “NA” in the subsequent statistical analysis. No significant differences were observed between systemic treatment response groups. Although non-LAR samples were mostly prevalent in pCR subgroup (84% of samples) and LAR samples were enriched in RP subgroup (47% of samples), the differences in TNBC subtypes distribution across pCR and RR subgroups were not statistically significant (*p* value 0.054).

**Table 1:** Summary descriptive table by groups of “Treatment.response”

	All patients *N* = 46	Complete response *N* = 26	Reduced response *N* = 20	*p*. overall
Age	53.5 [39.0;62.8]	51.0 [38.2;60.0]	60.0 [42.0;65.8]	0.1908
Ki67 status [%]	60.0 [40.0;70.0]	60.0 [46.2;70.0]	60.0 [37.5;70.0]	0.2444
Specimen purity [%]	55.0 [50.0;68.8]	50.0 [50.0;60.0]	60.0 [47.5;76.2]	0.2752
TNBC subtype LAR NON-LAR	13 (29.5%) 31 (70.5%)	4 (16.0%) 21 (84.0%)	9 (47.4%) 10 (52.6%)	

### Integration with TCGA breast cancer cohort

**Fig. 1:**
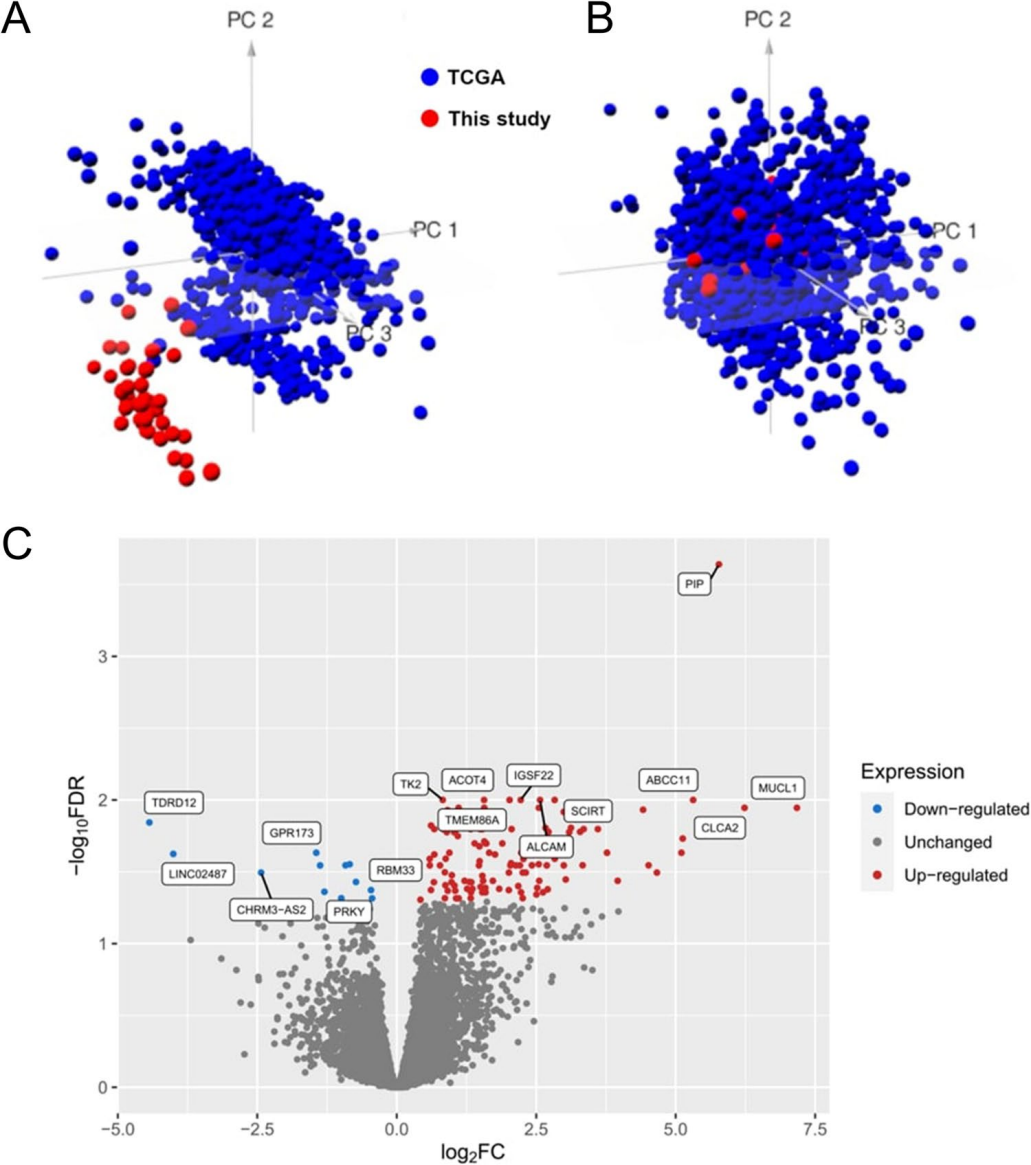
PCA of gene expression data and differential expression analysis. PCA was performed using gene expression data from FF (TCGA) or FFPE (This study). **A** Prior to batch correction, two different clusters were identified by PCA. **B** After batch correction, FF and FFPE samples formed one cluster. The variance in percentages accounted for by each principal component is shown on each axis. **C** Volcano plot resulting from differential expression analysis in RR versus CR patients. Significantly deregulated genes (abs. logFC ≥ 1.2, FDR ≤ 0.05) are depicted as blue dots (downregulated) and red dots (upregulated)

In order to further assess the quality of FFPE-extracted RNA and subsequent RNA-seq procedure, we decided to merge expression profiles of our samples with expression profiles of high quality fresh frozen (FF) samples from TCGA study. We used principal component analysis (PCA) of gene expression to assess differences and similarities between FFPE samples and TCGA FF samples (Fig. 1 A and B). Prior to batch correction, two different clusters corresponding to FF and FFPE samples were observed; however, after batch correction, FF and FFPE samples formed one coherent cluster. This suggests that the gene expression profiles obtained from FFPE samples were of quality comparable to FF samples.

### Gene expression analysis

**Fig. 2:**
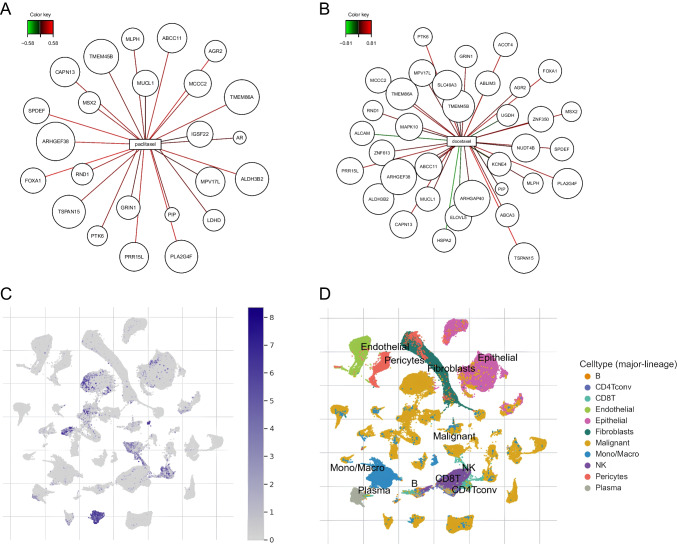
Further assessment differentially expressed genes. **A, B** Results of PLSR visualized by relevance associations network depicting DE genes associated (absolute correlation values ≥ 0.4) with sensitivity of breast cancer cell lines to paclitaxel (**A**) and docetaxel (**B**). **C, D** Exemplary visualization of expression of *MUCL1* gene in single-cell RNA-seq dataset obtained from breast cancer tumors (GSE161529). Note that the expression of *MUCL1* is present only in some tumor cells

Differential expression analysis of RR versus CR was performed with edgeR for the 24,237 genes. A total of 105 genes were significantly differentially expressed in comparison between RR versus CR patients with a significant level of FDR ≤ 0.05 and absolute fold change ≧ 1.2 (Fig. 1C) including six significantly downregulated genes and 99 significantly upregulated genes (see Supplementary Table 1). As revealed by PLSR analysis in breast cancer cell lines, out of 105 deregulated genes, 42 were associated with sensitivity to docetaxel, doxorubicin, paclitaxel, and/or cyclophosphamide (Fig. 2A, B and Supplementary Table 1). Next, we explored three primary breast cancer single-cell RNA-seq datasets, to select only those genes which are expressed in tumor malignant cells. We found that 24 out of 42 sensitivity-associated genes displayed intermediate or strong expression in breast malignant cells (Supplementary Table 1, Fig. 2A, B, C, D). Finally, we analyzed which of 24 genes were significantly associated with overall survival (OS) in TNBC TCGA dataset. We found that dichotomized expression (high/low) of nine out of 24 genes was significantly associated with OS in TNBC (Supplementary Table 1): *MUCL1*, *ABCC11*, *SPDEF*, *APOD*, *ARHGEF38*, *PRR15L*, *ABCA3*, *KCNE4*, and *CYB5A*. High expression of eight out of 10 genes was associated with shorter OS in TNBC except of *APOD* and *ARHGEF38* for which high expression was associated with longer OS in TNBC.

## Discussion

Neoadjuvant, systemic treatment of TNBC remains currently one of the standard therapeutic options preceding surgery. Its application brings the reduction of the primary tumor’s size and aims to eliminate lymph node and distant metastases (Asselain et al. 2018). At present, six therapeutic schemes are the preferred neoadjuvant regimens for TNBC:

- Taxel/docetaxel + adriamycin + cyclophosphamide (TAC)
- Docetaxel + cyclophosphamide (TC)
- Anthracycline + cyclophosphamide (AC)
- Cyclophosphamide + methotrexate + fluorouracil (CMF)
- Cyclophosphamide + adriamycin + fluorouracil (CAF)
- Cyclophosphamide + anthracycline + fluorouracil + paclitaxel/docetaxel (CEF-T)

Nevertheless, a number of TNBC cases display impressive primary tumor response to neoadjuvant chemotherapy, the major disadvantage of this therapeutic approach is chemoresistance which implicates a suboptimal efficacy in large percentage of patients (Echeverria, et al. 2019; Carbognin et al. 2015).

Chemoresistance, the insensitivity of cancer cells to chemotherapy, is the common event in cancer treatment, especially in TNBC (Cao et al. 2021). Some mechanisms leading to chemoresistance are well characterized and described, but still—because of its heterogeneity—there is an urgent need of further investigation and better understanding. Among these mechanisms are multidrug resistances connected with expression of ATP binding cassette (ABC) transporters, the activation of signaling pathways important for survival and cancer cell invasion, non-coding RNAs (ncRNAs) and their role in signaling and regulation of biochemical pathways, and cancer stem cells that overexpress various transporters and surface biomarkers that allow escaping from classical treatment (Cao et al. 2021). Considering the heterogeneous nature of triple-negative tumors, justifying the difficulties in TNBC treatment, new approaches suggest the personalization of the diagnostic and therapeutic strategies. Much research focuses on finding novel prognostic biomarkers for TNBC and the interactions between them to identify subtypes for further, potential targeted therapy. In terms of chemoresistance, gene expression profiling may help determine different TNBC subtypes with distinct sensitivity.

Some previous studies on transcriptomics analysis of breast cancer FFPE tissues revealed many differentially expressed genes. In patients with TNBC, a group of genes involved in mammary gland morphogenesis (*FSIP1*, *ADCY5*, *FSD1*, *HMSD*, *CMTM5*, *AFF3*, *CYP2A7*, *ATP1A2*, and *C11orf86*) was associated with prognosis. Three of them: *ADCY5*, *CYP2A7*, *ATP1A2* act in hormone-related pathways (Chen et al. 2020). Another study on non-metastatic BC treated with neoadjuvants revealed that lower expression of *CIQTNF3*, *CTF1*, *OLFML3*, *PLA2RI*, *PODN*, *KRTI5*, and *HLA-A* and overexpression of *TUBB* and *TCPI* was characteristic for patients with chemoresistance and with poor prognosis. These genes were encoded proteins from extracellular region and plasma membrane, the area of signal transduction (Barrón-Gallardo et al. 2022). Also, changes in gene expression for patients with estrogen-receptor–positive BC treated gradually with neoadjuvant and next adjuvant endocrine therapy were detected. Higher expression for *ER*, *HER2*, *GATA3*, *AKT*, *RAS*, and *p63*, genes that promote cell proliferation in resistant in comparison to sensitive tumors, was the expected result and consistent with endocrine-resistant mechanism (Xia et al. 2022).

In our study, after performing RNA-seq analysis, we identified nine genes involved in various cellular pathways, overexpressed in chemoresistant patients: *KCNE4*, *ABCC11*, *ABCA3*, *APOD*, *ARHGEF38*, *PRR15*, *CYB5A*, *SPDEF*, and *MUCL1*. There is a variation of functions among selected genes: ABC transporters, cytochromes, and transcripts controlling cell polarization. Different studies confirm that the abovementioned genes are strictly involved in cellular processes related to carcinogenesis.

*KCNE4* encoding potassium voltage-gated channel regulator was identified as differentially expressed between sensitive and resistant groups. *KCNE4* overexpression was reported as poor prognosis factor in various malignancies (Mano et al. 2022; Wu et al. 2022; Li et al. 2021). In the recent years, it was shown that different families of potassium channels are overexpressed in primary breast cancers. Being localized in the plasma membrane, ion channels could represent novel cancer biomarkers, and their detection might be easily performed by immunohistochemical and molecular techniques. Moreover, for the same reason, they represent a good potential target for therapy with specific drugs and antibodies (Ko et al. Sep. 2013). Different voltage-gated potassium channels are aberrantly expressed in TNBC (Lastraioli 2020). Previous studies show correlation between increased potassium channel expression and the development of metastases, nuclear grade, proliferation, and poor prognosis in breast cancer (Khaitan et al. Jul. 2009; Brevet et al. 2009; Jang et al. 2009). Interestingly, other potassium channels, such as Kv11.1 encoded by *KCNH2*, significantly reduce the metastatic spread of breast tumors in vivo while activated (Breuer, et al. 2019). Some studies indicate that some potassium channels are expressed in a variety of breast cancer cells but not in healthy tissue (Lansu and Gentile Jun. 2013; Iorio et al. Jul. 2018). Despite the pivotal role of potassium channels in the development of cancer, it is currently difficult to assign a specific mechanism for each ion channel in the proliferation, invasion, and metastasis of tumor cells (Li and Xiong 2011).

Our study revealed overexpression of two genes encoding family of ATP binding cassette (ABC) transporters proteins: *ABCA3* and *ABCC11*. A number of ABC transporters are strongly implicated in chemoresistance of numerous solid tumors, including breast cancer (Muriithi et al. May 2020). *ABCA3* is not well-characterized in terms of breast cancer comparing to *ABCC11*. It has been shown that increased *ABCA3* expression in breast cancer seems to be associated with poor prognosis. In the study of Schimanski et al., diminished *ABCA3* expression proved to be a significant, independent, and adverse risk factor for breast cancer recurrence (Schimanski et al. 2010). In other studies, overexpression of *ABCA3* increased chemoresistance (Overbeck et al. Jun. 2013) and conferred shorter relapse-free survival in different malignancies (Bartholomae et al. Feb. 2016). Several studies indicate that *ABCC11* gene, encoding multidrug-resistant protein-8, is expressed significantly more in breast cancer (including TNBC) (Yamada et al. Feb. 2013; Xu et al. 2017) and is associated with poor prognosis (Tsyganov et al. 2022; Nedeljković and Damjanović 2019). It has been shown that *ABCC11* confers resistance to anthracyclines, taxanes, mitoxantrone, and methotrexate (Sissung et al. Feb. 2010).

We observed increased expression of *APOD* in patients with partial response or disease progression. Apolipoprotein D (APOD) is a well-known, multifunctional glycoprotein that is expressed at 1000-fold higher levels in the cyst fluid of women with gross cystic disease of the breast, than in the plasma of the healthy women (Jankovic-Karasoulos et al. Jun. 2020). ApoD has been reported to be a marker of invasive BC, with promising, prognostic importance. Many studies show that ApoD expression is associated with poor BC survival outcome (Søiland et al. Feb. 2009; Díez-Itza, et al. 1994). It has been shown that ApoD expression may be downregulated via estrogen receptor signaling (Simard et al. 1990) and upregulated in the presence of tamoxifen (Harding et al. 2000). Patients with high ApoD expression in ER-positive breast cancers have a significantly poorer survival outcome than patients with ERα-positive breast cancers and low ApoD expression (Jankovic-Karasoulos et al. Jun. 2020). Studies show that patients with ApoD-negative tumors receiving tamoxifen therapy had a significantly better survival outcome than patients with ApoD-positive tumors (Søiland et al. 2009). The abovementioned studies, partially confirm our results that ApoD overexpression may be related to worse therapeutic outcomes in BC patients.

In our study, the overexpression of *PRR15* was observed in patients with reduced response to chemotherapy. Among a few reports regarding *PRR15* activity, there is an assumption that the abovementioned gene is involved in embryonic development, neurological disorders, and cancer (Lüönd et al. Jul. 2021; Yersal and Barutca Aug. 2014; Cserni, et al. 2021; Yin et al. Jun. 2020). The study of Wang et al. confirmed the role of *PRR15* in promotion of thyroid cancer and induction of changes in its microenvironment. It was observed that overexpression of *PRR15* correlates with increased infiltration of eosinophils and NK cells (Zhang et al. Mar. 2022). Some studies indicate that dysregulation of PRR15 expression is a negative prognostic factor in breast cancer, esophageal cancer, and some gastrointestinal malignancies (Cortazar et al. Jul. 2014; Holanek, et al. 2021). The role of PRR15 in breast cancer remains controversial. In the study of Guo et al., it was found that TNBC’s proliferation increases with the reduction of PRR15 expression. However, further studies are needed to assess the role of PRR15 expression changes in TNBC development (Minckwitz et al. Oct. 2013).

In patients with disease progression, we observed the overexpression of *ARHGEF38* (rho guanine nucleotide exchange factor 38). It is involved in regulation of catalytic activity, tumor cell polarization, and metastasis pathway. This gene, so far not well examined, was identified for the first time in 2021 as a possible biomarker of lung adenocarcinoma and lung squamous cell carcinoma (Chen and Dhahbi 2021). Recent studies indicate *ARHGEF38* as a novel predictive biomarker of aggressive prostate cancer as well (Liu et al. Jun. 2019; Sun 2021). Liu et al. observed that ARHGEF38 protein in lymph node metastasis patients was significantly higher than that in the non-metastatic patients, which may suggest that the high expression of *ARHGEF38* is more prone to distant metastasis (Liu et al. Jun. 2019). Our study reveals for the first time that *ARHGEF38* may be used as potential indicator of poor prognosis in TNBC patients.

Cytochrome b5 (encoded by *CYB5A*) detoxifies aromatic and heterocyclic amine mammary carcinogens found in cigarette smoke (Blanke et al. Oct. 2014)—one of the leading BC risk factors (Jones et al. 2017). We observed increased expression of *CYB5A* in TNBC patients presenting complete response. Up to date, function and regulating mechanisms of cytochrome b5 in breast cancer remain unknown. Recent studies confirmed that CYB5A reduces the oxidative stress levels, alters the apoptosis cascade, regulates ERK1/2 and Akt signaling pathways, and thus plays an important role in maintaining the balance of the redox system in cancer cells (Tong et al. 2022; Guo, et al. 2022). Nevertheless, the effects of overexpressed *CYTB5* are various in different breast cancer phenotypes, which is probably related to gene polymorphisms (Blanke et al. Oct. 2014). These observations provide useful information for understanding the multiple roles of cytochrome b5 and provide clues for further studies on personalized BC patient management.

*SPDEF* (SAM pointed domain containing ETS transcription factor) was first identified as an activator of prostate-specific antigen (PSA) (Oettgen et al. Jan. 2000), which can be detected in epithelial tissues including hormone‐regulated epithelia such as the prostate, breast, and ovary (Buchwalter et al. Jun. 2013). In cancer research, the role of *SPDEF* in BC depends on different subtypes and remains controversial. Several studies have demonstrated that high *SPDEF* expression promotes Luminal BC differentiation and correlates with poor OS in ER-positive breast cancer patients (Buchwalter et al. Jun. 2013; Sood et al. Nov. 2007; Sood et al. Jun. 2009). Our study showed that *SPDEF* expression levels were higher in TNBC patients with poor OS. The abovementioned observations exhibit *SPDEF* as a possible oncogenic factor. In contrast, the downregulation of *SPDEF* in invasive basal BC cell lines supports a tumor-suppressive role (Turner et al. Feb. 2007). Up-to-date studies proved that high expression of *SPDEF* may be utilized as prognostic factor for the poor OS in various BCs; nevertheless, further research on *SPDEF* expression patterns and molecular mechanisms underlying subtype-specific role of SPDEF are needed, to evaluate its role in the occurrence and development of multiple BC subtypes.

Small breast epithelial mucin (*MUCL1*) (also known as *SBEM*) gene is involved in invasion and metastasis of breast cancer via promoting epithelial‑to‑mesenchymal transition (Li et al. Aug. 2020). Based on its highly restricted mRNA expression in breast tissue and continued expression during breast tumorigenesis, *MUCL1* is an attractive tumor-associated antigen and a potential therapeutic target (Conley et al. 2016). Accordingly to these results, our research confirmed that *SBEM-MUCL1* was overexpressed in TNBC patients. In the study of Liu et al., it was shown that *SBEM* has the potential for predicting response to neoadjuvant chemotherapy in breast cancer. After 3 cycles’ neoadjuvant chemotherapy, *SBEM* expression levels were significantly downregulated in up to 58% breast cancer patients (Liu et al. Apr. 2010). Furthermore, some studies indicate *SBEM-MUCL1* as a marker for micrometastasis in breast cancer (Liu et al. Apr. 2010; Valladares-Ayerbes et al. Sep. 2009). Taken together, these data suggest a potential utility for therapeutic targeting of this protein in breast cancer, including TNBC.

## Conclusions

Our RNA-seq–based findings evidenced that 24 genes with tumor cell–specific expression in TNBC present different expression patterns in complete response/reduced response patients’ groups to neoadjuvant treatment in FFPE specimens. In addition, 10 out of 24 genes displayed prognostic properties. Given the malignant cell-specific expression of these genes, it should be further investigated for its potential to be translated into a predictive test.

## Supplementary Information

Below is the link to the electronic supplementary material.

Supplementary file1 (PDF 109 KB)

## Data Availability

The datasets generated and analyzed during the current study are available in the BioProject repository, with accession number: PRJNA964497; link: https://dataview.ncbi.nlm.nih.gov/object/PRJNA964497?reviewer=qcmbcn2489uf6c295p9urde9hf.
