# Correction to: The analysis of transcriptomic signature of TNBC—searching for the potential RNA‑based predictive biomarkers to determine the chemotherapy sensitivity

**DOI:** 10.1007/s13353-024-00902-y

**Published:** 2024-08-24

**Authors:** Stanislaw Supplitt, Pawel Karpinski, Maria Sasiadek, Lukasz Laczmanski, Dorota Kujawa, Rafal Matkowski, Piotr Kasprzak, Mariola Abrahamowska, Adam Maciejczyk, Ewelina Iwaneczko, Izabela Laczmanska

**Affiliations:** 1 Lower Silesian Oncology, Pulmonology and Hematology Center, Hirszfelda Sq. 12, 53‑413 Wroclaw, Poland; 2 Department of Genetics, Wroclaw Medical University, Marcinkowskiego 1, 50‑368 Wroclaw, Poland; 3 Laboratory of Genomics and Bioinformatics, Ludwik Hirszfeld Institute of Immunology and Experimental Therapy, Polish Academy of Sciences, Weigla 12, 53-114 Wroclaw, Poland; 4 Department of Oncology, Wroclaw Medical University, Hirszfelda Sq. 12, 53‑413 Wroclaw, Poland

**Correction to** **:** **Journal of Applied Genetics (2024)**

10.1007/s13353-024-00876-x

The article was initially published with the author names inverted, where the family name was listed first instead of the given name. This has now been updated here.

The original article has been corrected.

