# Supplementary material for: The analysis of transcriptomic signature of TNBC—searching for the potential RNA-based predictive biomarkers to determine the chemotherapy sensitivity
Source: J Appl Genet. 2024 May 9;66(1):171–82. doi: 10.1007/s13353-024-00876-x (PMC11761126; doi:10.1007/s13353-024-00876-x)
Supplement: Supplementary file 1 — Supplementary file1 (PDF 109 KB) [file 13353_2024_876_MOESM1_ESM.pdf]

Transcript	logFC	logCPM	FDR	SYMBOL	GENENAME
ENSG00000172551	7,172942489	7.5295480 1432365	0.0113424078362825	MUCL1	mucin like 1
ENSG00000121270	5,313371939	6.2612086 9199596	0.0099964587774512	ABCC11	ATP binding cassette subfamily C member 11
ENSG00000124664	3,34973727	4.0174077 9734226	0.0159528729051119	SPDEF	SAM pointed domain containing ETS transcription factor
ENSG00000189058	3,331979831	8.0451951 3931687	0.0285081162584976	APOD	apolipoprotein D
ENSG00000236699	2,719738498	4.5252663 4773914	0.0166738257781405	ARHGEF38	Rho guanine nucleotide exchange factor 38
ENSG00000172602	2,544669044	2.3532938 0593157	0.0280436499355728	RND1	Rho family GTPase 1
ENSG00000167183	1,588724228	4.0070240 0159055	0.0409348611222109	PRR15L	proline rich 15 like
ENSG00000167972	1,568433856	6.2432768 7043313	0.0113424078362825	ABCA3	ATP binding cassette subfamily A member 3
ENSG00000152049	1,548576749	3.2034791 7929853	0.0365579719804778	KCNE4	potassium voltage-gated channel subfamily E regulatory subunit 4
ENSG00000166347	1,330146901	4.9707827 3670033	0.0439646382337195	CYB5A	cytochrome b5 type A
ENSG00000106541	2,994440608	3.5739289 9462454	0.0285554828839684	AGR2	anterior gradient 2, protein disulphide isomerase family member
ENSG00000170017	2,568836739	7.2978960 4677402	0.0099964587774512	ALCAM	activated leukocyte cell adhesion molecule
ENSG00000132746	2,503864921	6.3518992 7902514	0.0455122703641609	ALDH3B2	aldehyde dehydrogenase 3 family member B2
ENSG00000169083	2,831859975	6.1469251 3295639	0.0256589386726342	AR	androgen receptor
ENSG00000012660	1,064906854	6.9273603 5235062	0.0484086616302818	ELOVL5	ELOVL fatty acid elongase 5
ENSG00000129514	3,768883537	5.0456564 4821764	0.0233132760223064	FOXA1	forkhead box A1
ENSG00000134202	2,547465808	4.4662584 4462642	0.0113424078362825	GSTM3	glutathione S-transferase mu 3
ENSG00000131844	1,303905604	6.3424015 0499654	0.0372330931300954	MCCC2	methylcrotonyl-CoA carboxylase subunit 2

ENSG00000115648	2,99316563	6.3180445 4355697	0.0121110420839795	MLPH	melanophilin
ENSG00000159763	5,775285211	4.7429874 8328102	0.000228887079746128	PIP	prolactin induced protein
ENSG00000099282	1,276241023	4.9983936 0954839	0.0143436795065847	TSPAN15	tetraspanin 15
ENSG00000109814	1,028768658	5.6502472 0850115	0.0167487126077347	UGDH	UDP-glucose 6-dehydrogenase
ENSG00000126803	2,058343081	3.8478859 4773042	0.0159528729051119	HSPA2	heat shock protein family A (Hsp70) member 2
ENSG00000151715	2,008665622	3.0156355 228844	0.0424072196986128	TMEM45B	transmembrane protein 45B
ENSG00000173210	1,736534362	4.2649354 271144	0.0156430337124322	ABLIM3	actin binding LIM protein family member 3
ENSG00000177465	1,565402268	1.4896303 6785503	0.0099964587774512	ACOT4	acyl-CoA thioesterase 4
ENSG00000124143	2,553421172	2.9103193 1411622	0.0365408744497144	ARHGAP40	Rho GTPase activating protein 40
ENSG00000162949	4,415496831	5.1339953 2329919	0.011700546265308	CAPN13	calpain 13
ENSG00000176884	2,621090186	2.9251680 3726009	0.0439646382337195	GRIN1	glutamate ionotropic receptor NMDA type subunit 1
ENSG00000179057	2,221797424	2.7369451 2095245	0.0099964587774512	IGSF22	immunoglobulin superfamily member 22
ENSG00000166816	1,569984407	2.4048245 5568879	0.0194484572541615	LDHD	lactate dehydrogenase D
ENSG00000109339	2,13438076	4.9604888 0742436	0.0285554828839684	MAPK10	mitogen-activated protein kinase 10
ENSG00000156968	3,28694059	5.4218963 5672021	0.0166738257781405	MPV17L	MPV17 mitochondrial inner membrane protein like
ENSG00000120149	2,422965863	2.2032963 7774486	0.0285081162584976	MSX2	msh homeobox 2
ENSG00000177144	1,04339719	3.8215578 6509481	0.0166738257781405	NUDT4B	nudix hydrolase 4B
ENSG00000168907	3,122320704	1.6840055 5253468	0.0156430337124322	PLA2G4F	phospholipase A2 group IVF
ENSG00000101213	1,494299777	3.5529218 2297168	0.0159528729051119	PTK6	protein tyrosine kinase 6

ENSG00000169026	1,323900992	3.3862958 3035672	0.0159528729051119	SLC49A3	solute carrier family 49 member 3
ENSG00000151117	2,022275177	4.9219010 6321581	0.0099964587774512	TMEM86A	transmembrane protein 86A
ENSG00000109906	3,02927557	5.2608054 2754758	0.0357950892402337	ZBTB16	zinc finger and BTB domain containing 16
ENSG00000256683	1,325712635	4.8329086 7804958	0.0483010416965925	ZNF350	zinc finger protein 350
ENSG00000176024	1,396977367	3.7812729 5567882	0.0200753655441141	ZNF613	zinc finger protein 613
ENSG00000137975	6,234189981	5.3254724 8518167	0.0113424078362825	CLCA2	chloride channel accessory 2
ENSG00000164120	5,126228112	4.6804281 9262446	0.0185657320741351	HPGD	15-hydroxyprostaglandin dehydrogenase
ENSG00000179593	5,103282762	6.1686021 3231835	0.0233132760223064	ALOX15B	arachidonate 15-lipoxygenase type B
ENSG00000227471	4,663380177	4.9130662 4225641	0.0320385115244291	AKR1B15	aldo-keto reductase family 1 member B15
ENSG00000134201	4,513750596	6.2143752 1702777	0.0285081162584976	GSTM5	glutathione S-transferase mu 5
ENSG00000235687	3,960609035	4.4077978 3192078	0.0365408744497144	LINC00993	long intergenic non-protein coding RNA 993
ENSG00000228793	3,60486415	3.8143002 743799	0.0159528729051119	LOC100507336	lncRNA Novel Transcript
ENSG00000181577	3,091870049	4.1372609 4322761	0.0167586377164096	C6orf223	chromosome 6 open reading frame 223
ENSG00000237686	2,830491107	3.3194498 4441615	0.0099964587774512	SCIRT	stem cell inhibitory RNA transcript
ENSG00000186204	2,705708332	1.6380150 5173219	0.041910079842348	CYP4F12	cytochrome P450 family 4 subfamily F member 12
ENSG00000172250	2,689025199	4.3526148 8654097	0.0285554828839684	SERHL	serine hydrolase like (pseudogene)
ENSG00000127954	2,666930638	5.9893864 7285263	0.0156430337124322	STEAP4	STEAP4 metalloreductase
ENSG00000070526	2,589877429	1.9898378 5941702	0.0200753655441141	ST6GALNAC1	ST6 N-acetylgalactosaminide alpha-2,6-sialyltransferase 1
ENSG00000166391	2,518765361	3.0491494 1493774	0.0233132760223064	MOGAT2	monoacylglycerol O-acyltransferase 2

ENSG00000159307	2,518300139	3.0099744 4616824	0.0409348611222109	SCUBE1	signal peptide, CUB domain and EGF like domain containing 1
ENSG00000226552	2,312735213	1.7570603 4187836	0.0320385115244291	LOC646804	alkylated DNA repair protein alkB homolog 8-like
ENSG00000183569	2,270074479	5.3933657 9594117	0.0257190797192355	SERHL2	serine hydrolase like 2
ENSG00000269235	2,258301629	4.5051730 1990713	0.0320385115244291	ZNF350-AS1	ZNF350 antisense RNA 1
ENSG00000259479	2,25807341	3.1039013 2637023	0.0483010416965925	SORD2P	sorbitol dehydrogenase 2, pseudogene
ENSG00000234593	2,232657894	2.5592739 919548	0.0233132760223064	KAZN-AS1	KAZN antisense RNA 1
ENSG00000165272	2,227991517	5.6939119 0258766	0.0439646382337195	AQP3	aquaporin 3 (Gill blood group)
ENSG00000245750	2,208614103	3.2054762 8528201	0.0285554828839684	DRAIC	downregulated RNA in cancer, inhibitor of cell invasion and migration
ENSG00000237773	2,187320313	2.6590193 6793273	0.0166738257781405	ENSG0000023 7773	lncRNA Novel Transcript
ENSG00000117507	2,178317649	2.6440821 5372723	0.0397562297198513	FMO6P	flavin containing dimethylaniline monoxygenase 6, pseudogene
ENSG00000231424	2,082331232	2.5301425 2149935	0.0256589386726342	FMO1-AS	Antisense To FMO1
ENSG00000117501	2,049137659	3.1331277 8300097	0.0275694131293162	MROH9	maestro heat like repeat family member 9
ENSG00000203721	2,037264766	2.9380025 9201794	0.0285554828839684	LINC00862	long intergenic non-protein coding RNA 862
ENSG00000187510	2,010061048	1.3060942 3699782	0.0320385115244291	PLEKHG7	pleckstrin homology and RhoGEF domain containing G7
ENSG00000254399	1,968637726	0.8911341 91824957	0.0439646382337195	GLYATL1P4	Glycine-N-Acyltransferase Like 1 Pseudogene 4
ENSG00000169919	1,891822575	6.7028817 6403722	0.0200753655441141	GUSB	glucuronidase beta
ENSG00000111052	1,836860518	2.4643132 842976	0.0415624296183697	LIN7A	lin-7 homolog A, crumbs cell polarity complex component
ENSG00000253406	1,787256985	1.7790173 0265386	0.020057471447205	ABLIM3-AS	Antisense To ABLIM3
ENSG00000283236	1,773226941	2.8558701 074571	0.0233132760223064	ENSG0000028 3236	Novel Zinc Finger Protein Pseudogene

ENSG00000240499	1,763544523	3.1814577 7880055	0.0285554828839684	CADPS2-AS	Antisense To CADPS2
ENSG00000116774	1,761755397	5.3222074 5066859	0.0239603561703782	OLFML3	olfactomedin like 3
ENSG0000228113	1,729290118	2.1214413 800315	0.0285554828839684	STEAP4-AS	Antisense To STEAP4
ENSG00000216921	1,723103913	2.0885043 5858747	0.0365408744497144	FAM240C	family with sequence similarity 240 member C
ENSG00000229056	1,6569729	1.2258398 5494309	0.0233132760223064	HECW2-AS1	HECW2 antisense RNA 1
ENSG00000005469	1,605665353	3.8202902 6709805	0.0200753655441141	CROT	carnitine O-octanoyltransferase
ENSG00000186395	1,592774335	3.7412525 2563384	0.0200753655441141	KRT10	keratin 10
ENSG00000239627	1,591648505	1.1843614 7431882	0.0439646382337195	RPL12P20	ribosomal protein L12 pseudogene 20
ENSG00000076258	1,535297886	3.2911177 9293189	0.0159528729051119	FMO4	flavin containing dimethylaniline monoxygenase 4
ENSG00000095321	1,534108807	4.9136338 0718268	0.0336959822674017	CRAT	carnitine O-acetyltransferase
ENSG00000250508	1,533031205	2.1185737 0446693	0.0439646382337195	LINC02701	long intergenic non-protein coding RNA 2701
ENSG00000101417	1,503181814	4.7311066 47737	0.0403163968106867	PXMP4	peroxisomal membrane protein 4
ENSG00000246763	1,485188775	0.9678622 58744001	0.0266812355572431	RGMB-AS1	RGMB antisense RNA 1
ENSG00000116171	1,482653682	6.4963571 3703411	0.0210471195186337	SCP2	sterol carrier protein 2
ENSG00000084444	1,479369077	4.1810072 5783331	0.0156430337124322	FAM234B	family with sequence similarity 234 member B
ENSG00000099194	1,474146849	8.7565049 1449109	0.0285554828839684	SCD	stearoyl-CoA desaturase
ENSG00000226491	1,417894453	1.8060319 6708133	0.0409348611222109	FTOP1	FTO Pseudogene 1
ENSG00000137501	1,369457813	6.0253634 4350676	0.0154427340454485	SYTL2	synaptotagmin like 2
ENSG00000164251	1,356530093	2.7445179 9417818	0.0415624296183697	F2RL1	F2R like trypsin receptor 1

ENSG00000236830	1,336044577	4.5526904 7747322	0.0373812306880485	CBR3-AS1	CBR3 antisense RNA 1
ENSG00000259575	1,274741758	1.9948863 3383831	0.0318876596717267	RORA-AS	Antisense To RORA
ENSG00000248429	1,262456715	3.4489827 8850213	0.0415624296183697	GASK1B-AS1	GASK1B antisense RNA 1
ENSG00000260417	1,217485995	4.0193383 6513618	0.0484086616302818	ENSG0000026 0417	lncRNA Novel Transcript
ENSG00000164403	1,216359619	3.9072036 5816406	0.0365579719804778	SHROOM1	shroom family member 1
ENSG00000099725	-1,296146806	1.7185349 6600054	0.043568075456161	PRKY	protein kinase Y-linked (pseudogene)
ENSG00000129167	-1,37346914	1.2536590 4137051	0.0285554828839684	TPH1	tryptophan hydroxylase 1
ENSG00000184194	-1,44248087	3.6975682 6432047	0.0233132760223064	GPR173	G protein-coupled receptor 173
ENSG00000233355	-2,431174677	4.7277078 6796004	0.0320385115244291	CHRM3-AS2	CHRM3 antisense RNA 2
ENSG00000203688	-4,00536892	3.6165660 5176694	0.0237432886064757	LINC02487	long intergenic non-protein coding RNA 2487
ENSG00000173809	-4,434362696	3.5659650 7106661	0.0143436795065847	TDRD12	tudor domain containing 12

Supplementary Table 1.

Significantly differentially expressed genes in comparison between RR versus CR patients
